# Supplementary material for: Increased Vulnerability to Ferroptosis in FUS-ALS
Source: Biology (Basel). 2024 Mar 26;13(4):215. doi: 10.3390/biology13040215 (PMC11048265; doi:10.3390/biology13040215)
Supplement: Supplementary file 1 [file biology-13-00215-s001.zip › biology-2902255-supplementary.pdf]

Supplementary Figure S1: Mitochondrial depolarization observed in FUS-mutated cells.

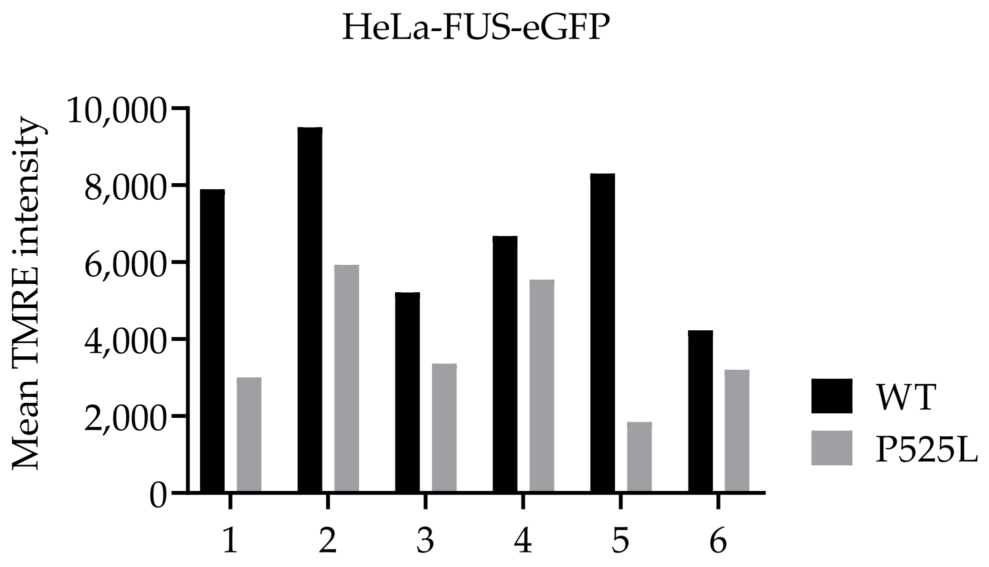

Individual values of TMRE from 6 different experiments. The mean TMRE signal intensity was quantified using MitoTracker deep red as counterstain for detection of mitochondria. Data are from 6 independent biological replicates (N=6).

Supplementary Figure S2: Whole blots of different proteins of Figure 4

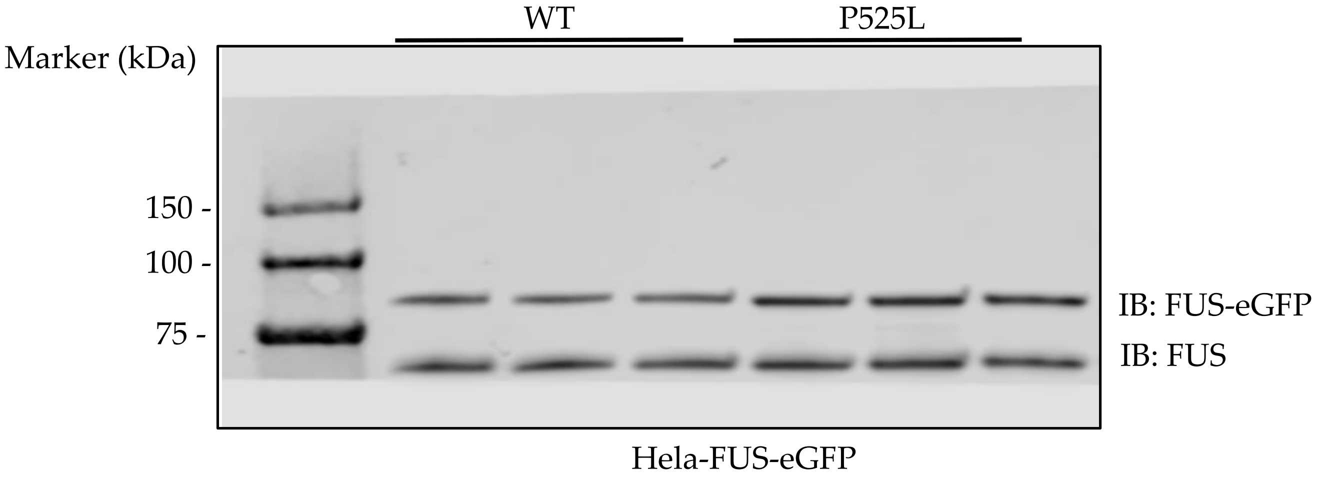

The initial three bands correspond to HeLa-WT-FUS, while the subsequent three represent HeLa-P525-FUS. The blot has been cut below the expexted size of the FUS prior to the staining, since it was planned for other antibody stainings. Both the upper and lower sections of the blot were subsequently treated with a different antibody for staining.

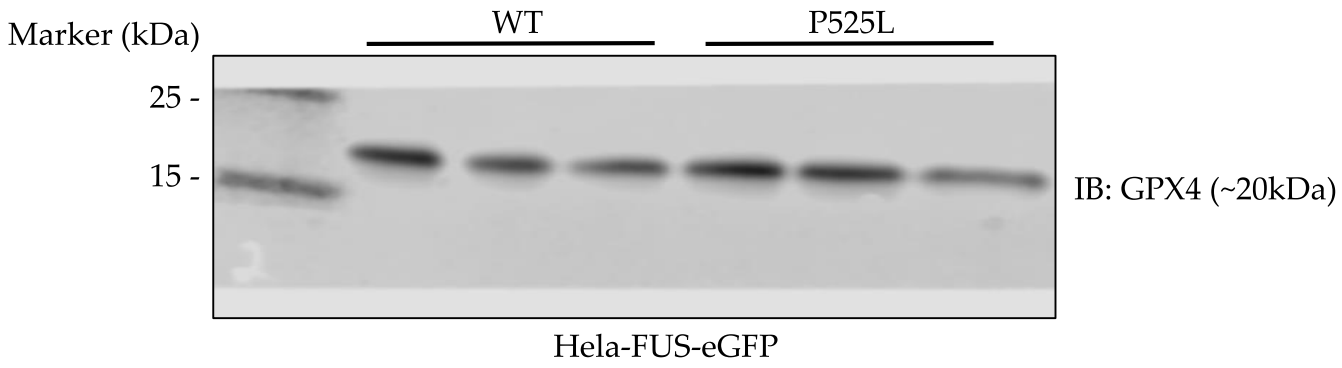

The initial three bands correspond to HeLa-WT-FUS, while the subsequent three represent HeLa-P525-FUS. The blot has been cut below the expexted size of the GPX4 prior to the staining, since it was planned for other antibody stainings. Both the upper and lower sections of the blot were subsequently treated with a different antibody for staining.

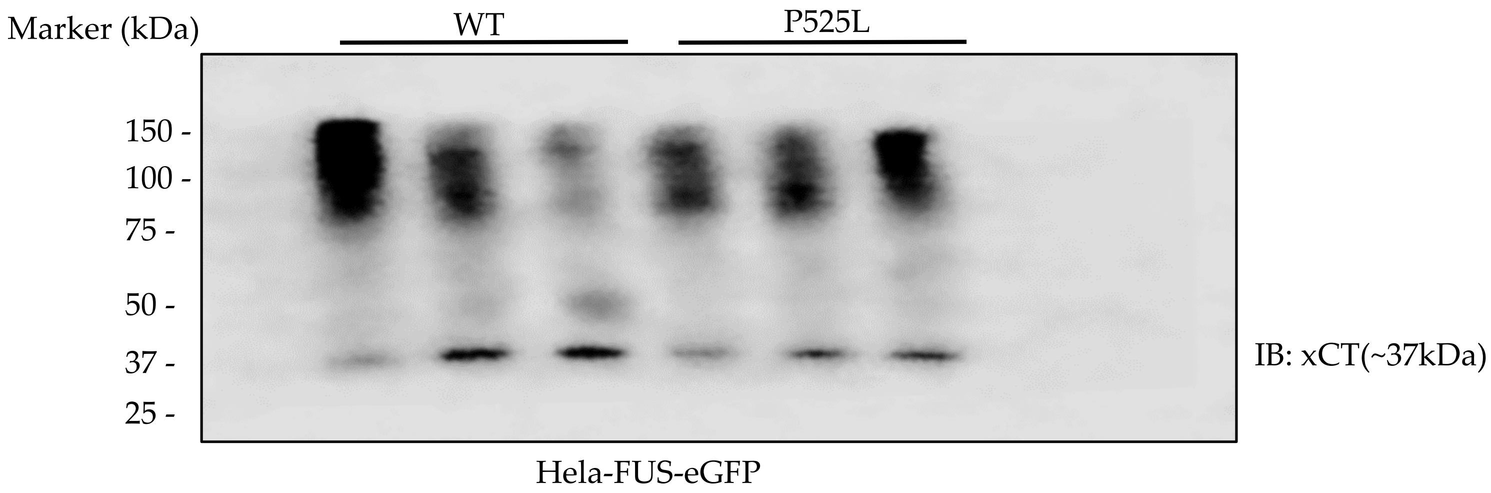

The initial three bands correspond to HeLa-WT-FUS, while the subsequent three represent HeLa-P525-FUS. The blot has been cut below the expexted size of the xCT prior to the staining, since it was planned for other antibody stainings. Both the upper and lower sections of the blot were subsequently treated with a different antibody for staining.

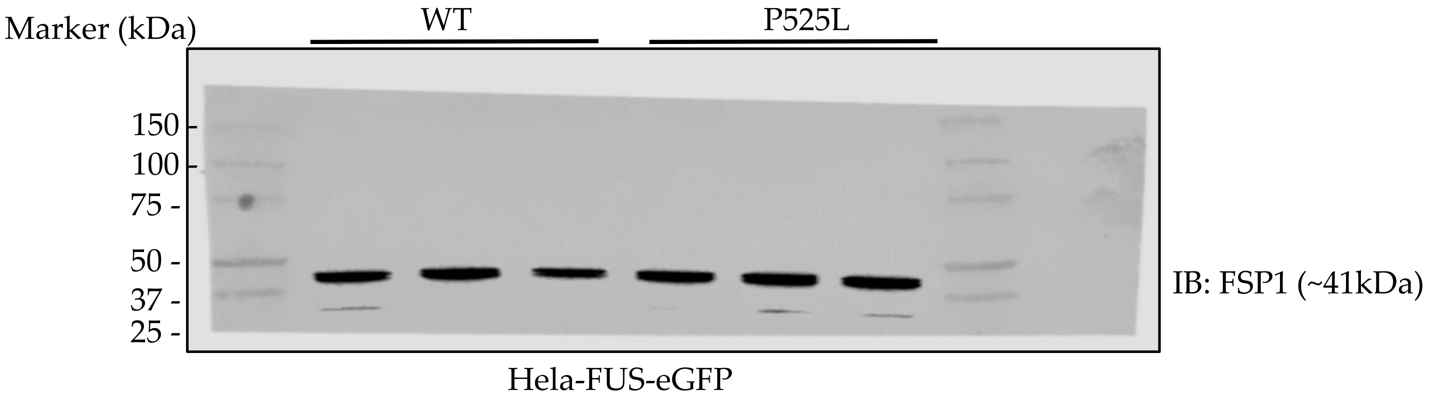

The initial three bands correspond to HeLa-WT-FUS, while the subsequent three represent HeLa-P525-FUS. The blot has been cut below the expexted size of the FSP1 prior to the staining, since it was planned for other antibody stainings. Both the upper and lower sections of the blot were subsequently treated with a different antibody for staining.

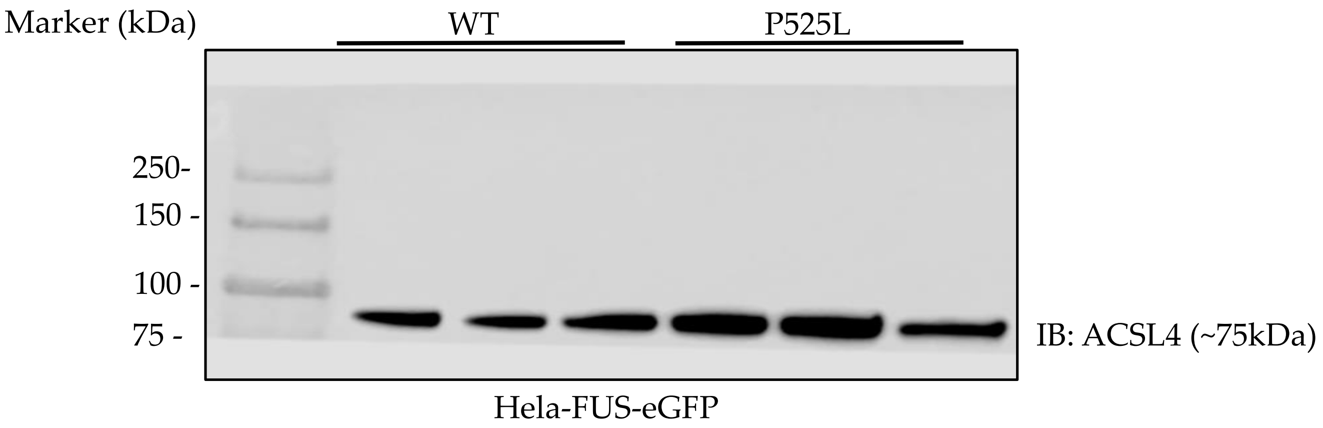

The initial three bands correspond to HeLa-WT-FUS, while the subsequent three represent HeLa-P525-FUS. The blot has been cut below the expexted size of the ACSL4 prior to the staining, since it was planned for other antibody stainings. Both the upper and lower sections of the blot were subsequently treated with a different antibody for staining.

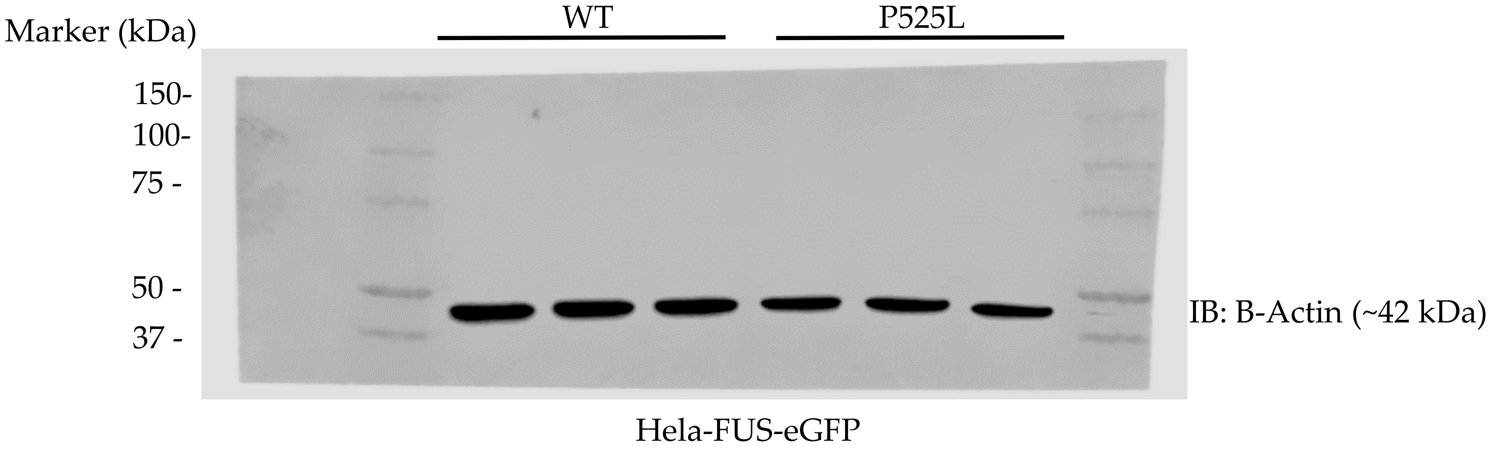

The initial three bands correspond to HeLa-WT-FUS, while the subsequent three represent HeLa-P525-FUS. The blot has been cut below the expexted size of the B-Actin prior to the staining, since it was planned for other antibody stainings. Both the upper and lower sections of the blot were subsequently treated with a different antibody for staining.
